# Temozolomide-Perillyl alcohol conjugate impairs Mitophagy flux by inducing lysosomal dysfunction in non-small cell lung Cancer cells and sensitizes them to irradiation

**DOI:** 10.1186/s13046-018-0905-1

**Published:** 2018-10-16

**Authors:** Minghui Chang, Xingguo Song, Xinran Geng, Xingwu Wang, Weijun Wang, Thomas C. Chen, Li Xie, Xianrang Song

**Affiliations:** 1 School of Medicine and Life Sciences, University of Jinan, Shandong Academy of Medicine Science, Jinan, Shandong People’s Republic of China; 2 Department of Clinical Laboratory, Shandong cancer hospital affiliated to Shandong University, Shandong Academy of Medical Sciences, 440 Ji-Yan Road, Jinan, 250117 Shandong Province People’s Republic of China; 3 Shandong Provincial Key Laboratory of Radiation Oncology, Shandong cancer hospital affiliated to Shandong University, Shandong Academy of Medical Sciences, 440 Ji-Yan Road, Jinan, 250117 Shandong Province People’s Republic of China; 4 Maternity & Child Care Center of Dezhou, Dongdizhong Street 835#, Decheng District, Dezhou, Shandong People’s Republic of China; 5 Departments of Neurological Surgery, and Pathology, University of Southern California, California, Los Angeles USA

**Keywords:** TMZ-POH, Mitophagy, RAB7A, NSCLC

## Abstract

**Background:**

Temozolomide-perillyl alcohol conjugate (TMZ-POH), a novel Temozolomide (TMZ) analog developed based on the conjugation of TMZ and perillyl alcohol (POH), displayed strong anticancer potency in multiple cancer types. In this study, we aimed to clarify the relationship between TMZ-POH and autophagy, and explore the underlying mechanisms involved in.

**Methods:**

The proteins involved in autophagy, mitochondrial fission, lysosomal function and membrane traffic were detected by western blots; Autophagosome, mitochondria and lysosome were visualized by transmission electron microscope (TEM) and immunostaining; Apoptosis analysis and fluorescence probe detection were applied by flow cytometry.

**Results:**

TMZ-POH blocked mitophagy flux although the number of autophagosomes which colocalized with mitochondria in the cells was increased via inducing lysosomal dysfunction as evidence from impaired lysosomal acidification, maturation and hampered autophagosome- lysosome fusion, which largely depended on its downregulation on the small GTPase RAB7A via mevalonate pathway. More importantly, our data demonstrated TMZ-POH sensitized cancer cell to irradiation induced apoptosis.

**Conclusions:**

Temozolomide-perillyl alcohol conjugate impairs mitophagy flux by inducing lysosomal dysfunction in Non-Small Cell Lung Cancer (NSCLC) cells and sensitizes them to irradiation, thereby proposing TMZ-POH as a potential radiosensitizer.

**Electronic supplementary material:**

The online version of this article (10.1186/s13046-018-0905-1) contains supplementary material, which is available to authorized users.

## Background

Temozolomide-perillyl alcohol conjugate (TMZ-POH), a novel temozolomide (TMZ) analog, is developed based on the conjugation of temozolomide (TMZ), a clinically approved alkylating agent, and perillyl alcohol (POH), a naturally occurring monoterpene which has the amazing capability to enhance the cytotoxicity of TMZ in several tumors [1]. Previous studies had revealed that TMZ-POH displayed stronger anti-cancer potency than its individual constituents to several types of malignancy such as glioma [2], triple-negative breast cancer (TNBC) [3], non-small cell lung cancer (NSCLC) [4] and nasopharyngeal carcinoma (NPC) [5]. The beneficial effects of TMZ-POH depend on it-induced reactive oxygen species (ROS) accumulation, the key contributor to its anti-tumor activities, which triggers cell cycle-dependent DNA damage and G_2_M arrest as well as downstream signal activation, and finally cell death [4].

Recently, accumulating evidence have demonstrated ROS accumulation is associated with alteration of mitochondrial structure and shape through mitochondrial dynamics [6]. Accumulated ROS can cause mitochondrial damage and imbalance between mitochondrial fusion and fission. This imbalance can affect mitochondrial metabolism and functions, and initiates some protective mechanisms to removal dysfunctional mitochondria, such as autophagy, a conserved eukaryotic catabolic reaction that occurs continuously to remove and recycle damaged proteins and organelles, termed “mitophagy” [7]. Dysfunctional mitochondria are delivered by autophagosome into lysosome at the end stage of mitophagy, which involves lysosome maturation and fusion with autophagosome [8]. The most important biochemical feature of the lysosome is its acidic lumen, whose acidification is maintained by the lysosomal membrane, containing more than 20 lysosomal membrane proteins, including lysosome-associated membrane protein (LAMP) 1 and 2. Notably, LAMP1 and 2 are responsible to the fusion between autophagosomes and lysosomes. Their deficiency arrests phagosomal maturation and blocks autophagosome-lysosome fusion due to the reduced ability to move toward the microtubule-organizing center [9].

Another mechanism involved in lysosome function and its fusion with autophagosome is small GTPases such as Ras-associated binding protein 7 (RAB7A), the better characterized of the two small GTPases enriched on the late endosome (LE)/lysosome pool present in the perinuclear region of the cell near the microtubule organizing center [10]. The maturation from early to late endosomes is accompanied by the transition from association with RAB7A, which is also known as “RAB conversion” [11]. Besides,RAB7A recruits its effectors RILP [12] to promote fusion with endocytic, phagocytic vesicle, herein RAB7A serves as a master regulatory component for the biogenesis of autophagosomes, lysosomes and other lysosome-related organelles [13]. In addition, RAB7A activation depends on its prenylation by mevalonate pathway [14], which allows for the attachment of the RAB7A proteins into the lipid bilayer of the organelle, consequently for the correct targeting and function of RAB7A [15].

Previous studies have established the association between chemotherapy with TMZ and autophagy [16, 17]. Interestedly, when autophagy was prevented at an early stage by 3-methyladenine (3-MA), the antitumor effect of TMZ was suppressed, whereas bafilomycin A1 (Baf.A1) that prevents autophagy at a late stage by inhibiting lysosome acidification and its fusion with autophagosome [18], sensitized tumor cells to TMZ by inducing apoptosis, indicating the chemotherapy efficacy of TMZ depends on it induced autophagic flux status.

In this study, we aimed to clarify the relationship between TMZ-POH and autophagy, and explore the underlying mechanisms involved in. We found that TMZ-POH blocked mitophagy flux although the number of mitophagosomes in cells was increased. TMZ-POH impaired lysosomal acidification and maturation, and hampered autophagosome-lysosome fusion, which largely depended on its downregulation on the small GTPase RAB7A. More importantly, our data demonstrated TMZ-POH sensitized cancer cell to irradiation induced apoptosis, thereby proposing TMZ-POH as a potential radiosensitizer.

## Methods

### Cell lines and chemicals

Human non-small cell lung cancer (NSCLC)-derived cell lines A549, SPC-A1, NCI-H460 and NCI-H520 were purchased from American Type Culture Collection (Manassas, VA, USA) and China Center for Type Culture Collection (Wuhan, China). All these cells were grown in Dulbecco’s modified Eagle’s medium (DMEM, Gibco, Invitrogen, Carlsbad, CA, USA) supplemented with 10% fetal bovine serum (Gibco, Invitrogen) and antibiotics (penicillin/streptomycin, 100 U/ml) at 37 °C in 5% CO_2_.

TMZ-POH and Perillyl alcohol (POH) were provided by Neonc Technologies, Inc. (Los Angeles, USA) and diluted with DMSO to make stock solutions of 100 mM. Temozolomide (TMZ), 3-Methyladenine (3-MA), baflomycin A1 (Baf.A1), carbonyl cyanide m-chlorophenylhydrazone (CCCP), Nicotinamide (NAM), mevalonolactone (MVL), geranylgeraniol (GGOH), catalase (CAT) and N-acetyl-L-cysteine (NAC), Earle’s Balanced Salt Solution (EBSS) (Sigma-Aldrich, Shanghai, China) were dissolved in DMSO or deionized water dependently; In all cases of cell treatment, the final DMSO concentration in the culture medium never exceeded 0.5%. Stock solutions of all drugs were stored at − 20 °C.

### Adenovirus infection

Recombinant adenoviral vector carrying the human mRFP-GFP-LC3 gene was purchased from HanBio (Wuhan, China). Cells were plated in 12-well plates at a density of 1 × 10^4^ cells per well. Cells were infected at an MOI of 2 with GFP-mRFP-LC3 gene for 24 h. After washing with PBS twice, cells were treated with TMZ-POH for another 48 respectively.

### Autophagy/mitophagy induction and inhibition

For non-selective autophagy induction, cells were washed 3 times with pre-warmed PBS and then incubated with EBSS medium (Sigma-Aldrich) at 37 °C in 5% CO_2_ for 2 h. For mitophagy induction, cells were incubated with CCCP (10 μM) or NAM (5 mM) for 48 h respectively. For autophagy inhibition, cells were treated with 3-MA (1 mM), or Baf.A1 (10 nM) for 48 h respectively.

### Flow cytometry for fluorescence probe detection

Cells following the above treatment were loaded different fluorescence probes including Mito-Tracker Green (MTG) and Lyso-Tracker Red (LTR) (Beyotime, Beijing, China) for the indicated time as described above. After washing 3 times with PBS, the florescence intensities were measured by a FACS Calibur instrument (Becton Dickinson, USA) and the data were analyzed using FlowJo Software 7.6 (Treestar, Inc., CA).

### Immunostaining

For immunostaining, cells were fixed with 4% paraformaldehyde and permeabilized with 0.1% Triton X-100 for 15 min. After incubation for 1 h with the following primary antibodies: antibodies against human anti-LC3B, anti-SQSTM1, anti-COX-IV, anti-TOM20 (CTS, Cell Signaling Technology, Danvers, MA, USA), anti-EEA1, anti-Parkin (Abcam, Shanghai, China), anti-LAMP1 (Santa Cruz, California, USA) and washing with PBS, cells were incubated for 1 h with Alexa 488-conjugated (1:1000) or Alexa 555-conjugated (1:500) (Abcam) secondary antibodies, washed with PBS. Nuclei were stained by 4′, 6-diamidino-2-phenylindole (DAPI) (Beyotime) for 3 min. Microscopy was done on a confocal laser microscopy (LSM780, Carle Zeiss, Germany) or DeltaVision microscopy (GE Healthecare Life Science, USA).

For quantification of the number of autophagosomes (diameters 0.3–1.0 μM) and SQSTM1 positive dots (diameters 0.3–1.0 μM) and EEA1 positive dots (diameters 0.1–1.0 μM), at least five cells were randomly chosen, all eligible puncta were recorded and analyzed using Fiji ImageJ software [19]. Quantification of GFP and mRFP fluorescence intensity, and colocalization between two different signals were recorded and analyzed using Fiji ImageJ software.

### Apoptosis analysis

Apoptosis was evaluated by using the Annexin V-FITC Apoptosis Detection Kit (BD Biosciences Pharmingen, San Diego, USA) according to the description provided by the manufacturer. 1.5 × 10^5^ cancer cells grown in six well plates overnight were exposed to indicated drug treatment or 10 Gy irradiation (X-RAD 225, Radsource, Buford, USA) for indicated time, and then the cells were trypsinized, collected and stained with FITC-Annexin V & Propidum Iodide (PI) for 15 min in the dark. The stained cell population were determined using by a FACS Calibur instrument (Becton Dickinson) and the data were analyzed using FlowJo Software 7.6 (Treestar).

### Transmission electron microscope (TEM)

Cells were fixed in TEM stationary solution (2.5% glutaraldehyde in 0.2 M HEPES, G1102, Wuhan servicebio technology) at 4 °C for 4 h, rinsed in PBS, and then embedded in 4% agarose. After fixation in 1% osmium tetroxide for 2 h, the specimens were dehydrated using alcohol and embedded in polybed 812 resin (90529–77-4, SPI). After polymerization at 60 °C for 48 h, ultrathin sections were prepared with the Leica Ultracutuct slicer (Leica EM UC6, Germany), stained with uranyl acetate and lead citrate, and analyzed using TEM (HT7700, HITACHI). Count, measure and analysis on TEM picture were carried out using Fiji ImageJ software.

### Preparation of the cytoplasmic and mitochondrial fractions

Mitochondrial and cytoplasmic proteins were collected using a cell Mitochondria Isolation kit (Beyotime) in accordance with the manufacturer’s instructions. Briefly, cells were harvested and washed twice with ice-cold PBS, incubated in Lysis Buffer, and then transferred to glass homogenizers and homogenized in the ice for 30–40 times, centrifuged at 1200×g for 5 min to remove any nuclei, membrane fragments and unbroken cells, and the supernatant was further centrifuged at 15,000×g for 10 min at 4 °C. The resulting supernatant contained the cytoplasmic fraction and the pellet contained the mitochondrial fraction. The mitochondrial pellet was further resuspended in a mitochondrial lysis buffer at 4 °C.

### Western blots

Fifty μg quantity of protein was separated on SDS-PAGE and transferred onto PVDF membranes (Millipore, Billerica, MA, USA). Membranes were then blocked with 5% evaporated skimmed milk (Bio-rad, USA) in Tris-buffered saline (50 mM Tris-HCl, pH 7.5, 150 mM NaCl) containing 0.1% Tween-20 for 1 h, and probed overnight at 4 °C with the following primary antibodies: antibodies against human LC3B, SQSTM1/P62, HSP60 (1:1000; Cell Signaling Technology, CST), antibody against RAB7A, RILP, EEA1, LAMP2, ACTB (1:1000, Proteintech, China), antibody against LAMP1 (1:500; Santa Cruz, USA), followed by incubation with horseradish peroxidase coupled secondary anti-mouse or anti-rabbit antibodies (Proteintech) for 1 h at room temperature. The protein bands were visualized using ECL blotting detection reagents (Bio-rad, USA), and developed and fixed onto x-ray films. ACTB was served as a loading control.

### RAB7A activity assay

RAB7A activity was determined using a RAB7A activity assay kit (NewEast Biosciences, King of Prussia, PA) according to manufacturer recommended protocol. Briefly, lysates containing equal amounts of total proteins were incubated with a mouse monoclonal antibody recognizing GTP bound RAB7A specifically. The bound active RAB7A was pulled down by protein A/G agarose and detected by a rabbit polyclonal anti-RAB7A antibody.

### Statistical analysis

Statistical significance was evaluated with data from at least three independent experiments or at least five duplicates. GraphPad Prism 6.02 (GraphPad Software, San Diego, CA, USA) was used for data analysis. Statistical analysis was carried out using Student *t*-test and *ANOVA.* Data are presented as the mean ± SD. Significance was established at *P* < 0.05.

## Results

### TMZ-POH induces autophagosome formation

**Fig. 1:**
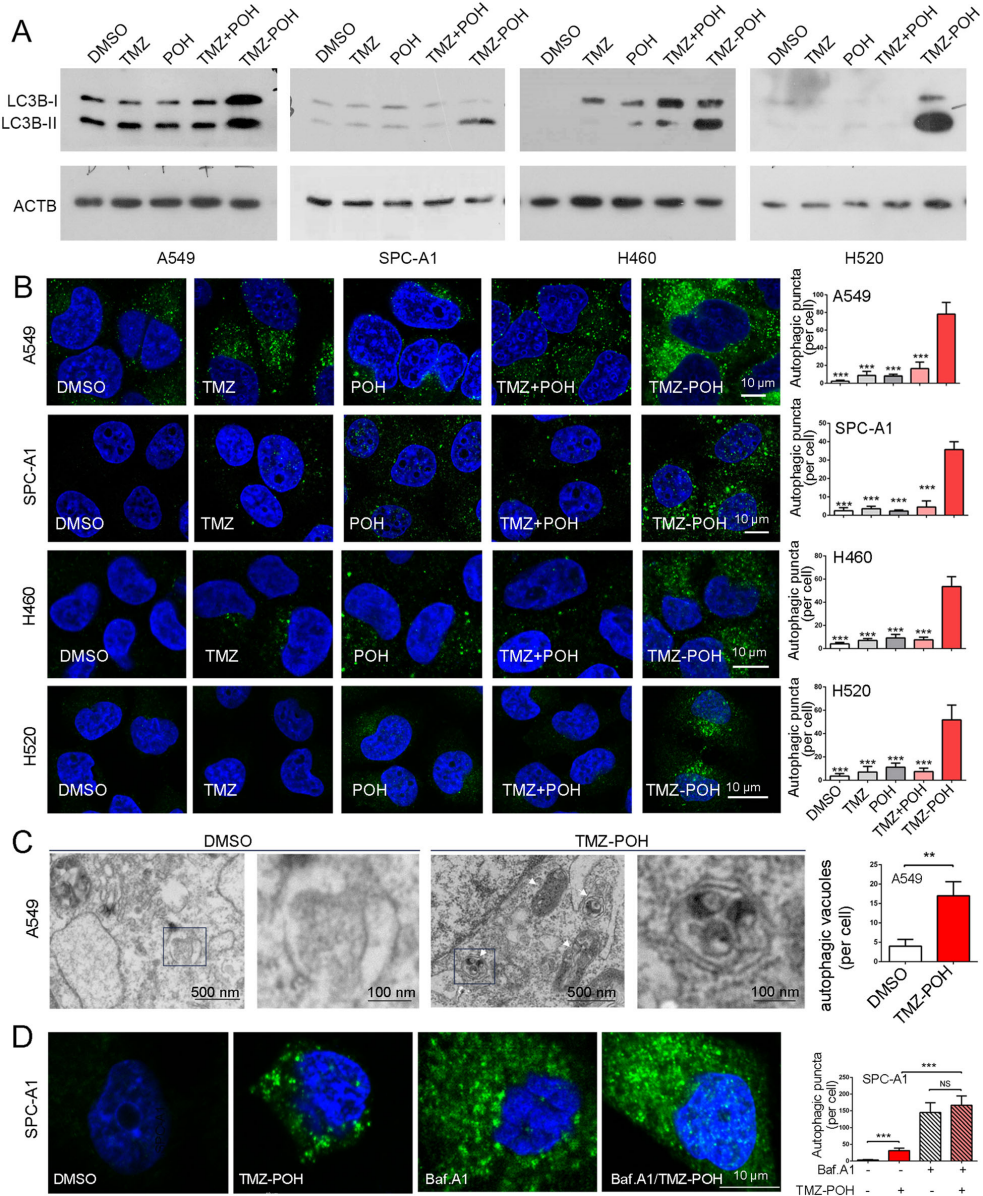
TMZ-POH induces autophagosome formation. **a**, **b** Cells were treated with 100 μM TMZ, POH, TMZ + POH, TMZ-POH or DMSO respectively for 48 h. **a** Western blot analysis demonstrated LC3B and ACTB expression in above drug-treated A549, SPC-A1, H460 and H520 cells; (**b**) The above drug-treated cells were inspected under confocal laser microscopy to detect LC3B puncta by immunofluorescence. LC3B puncta number per cell was quantified using the Fiji Image J program; (**c**) Autophagic vacuoles in A549 cells treated with 100 μM TMZ-POH or DMSO were observed by transmission electron microscopy (TEM). The arrow indicates autophagic vacuoles. Number of autophagic vacuoles were calculated using Fiji Image J software. **d** SPC-A1 cells treated with 100 μM TMZ-POH or DMSO were inspected under confocal laser microscopy to detect LC3B puncta by immunofluorescence in the presence or absence of Baf.A1. The results shown are means ±SD, **p < 0.005, ***p < 0.001, NS = no significance

To illuminate the effect of TMZ-POH on cell autophagy, four NSCLC cell lines including A549, SPC-A1, NCI-H460 (H460) and NCI-H520 (H520) were employed and subjected to 100 μM TMZ, POH, TMZ plus POH (TMZ + POH) and TMZ-POH, respectively. As shown in Fig. 1a and Additional file 1: Figure S1A, autophagy was activated significantly when treated by TMZ-POH rather than other drugs, as evidence from the increases in the amount of LC3B-II, the important markers of autophagy [20] in all detected cell lines, indicating autophagy activation by TMZ-POH is universal independent of cell type. Next, we checked the formation of autophagosomes by staining endogenous LC3B. We found that TMZ-POH treatment increased intracellular autophagosomes compared to its individual constituents and their combination, as demonstrated by accumulation of LC3B-positive spot-like structures in above drug treated four NSCLC cells (Fig. 1b). In addition, TMZ-POH-induced autophagosome accumulation appeared to be concentration-dependent, as the number of autophagic puncta increased with the concentration of TMZ-POH (Additional file 1: Figure S1B). Furthermore, this phenomenon was further confirmed by transmission electron microscope (TEM). Clearly, TMZ-POH treatment significantly increased intracellular autophagic vacuoles shown as double membrane vesicles with visible cytoplasm contents (Fig. 1c).

To rule out the possibility that TMZ-POH promoted excessive autophagic degradation which led to the failure in autophagosome accumulation, we treated cells combined with Baf.A1, a lysosomal inhibitor leading to accumulation of autophagic vacuoles [18]. As shown in Fig. 1d and Additional file 1: Figure. S1C, we found that in absence of Baf.A1, the number of intracellular autophagic puncta (Fig. 1d) and the amount of LC3B-II (Additional file 1: Figure S1C) were significantly increased when treated with TMZ-POH, whereas upon Baf.A1 treatment to block autophagic flux, these differences caused by TMZ-POH were eliminated, indicating a promotion of excessive autophagic degradation was not involved in the process that TMZ-POH induced autophagosome accumulation.

Induction of autophagy can occur through PI3K-AKT pathway which then phosphorylates mTOR [21]. mTOR inhibits autophagy by targeting autophagy related protein (ATG) 13 [22], and in turn transmits signals to downstream effectors such as autophagy-related gene beclin 1 (BECN1). mTOR functions by directly phosphorylating the key translation regulators p70 ribosomal S6 kinase (P70S6K), leading to an increase in translation of a subset of mRNAs [21]. Therefore, we detected whether TMZ-POH accumulated autophagosome dependent on mTOR signaling. Unexpectedly, TMZ-POH seemed to have no obvious effects on phosphorylation of mTOR itself and its specific substrate P70S6K, and the expression of its downstream effector BECN1 in SPC-A1 and NCI-H460 cells, indicating TMZ-POH-induced autophagosome formation is mTOR independent (Additional file 1: Figure S1D and E).

### TMZ-POH leads to mitochondria fission

**Fig. 2:**
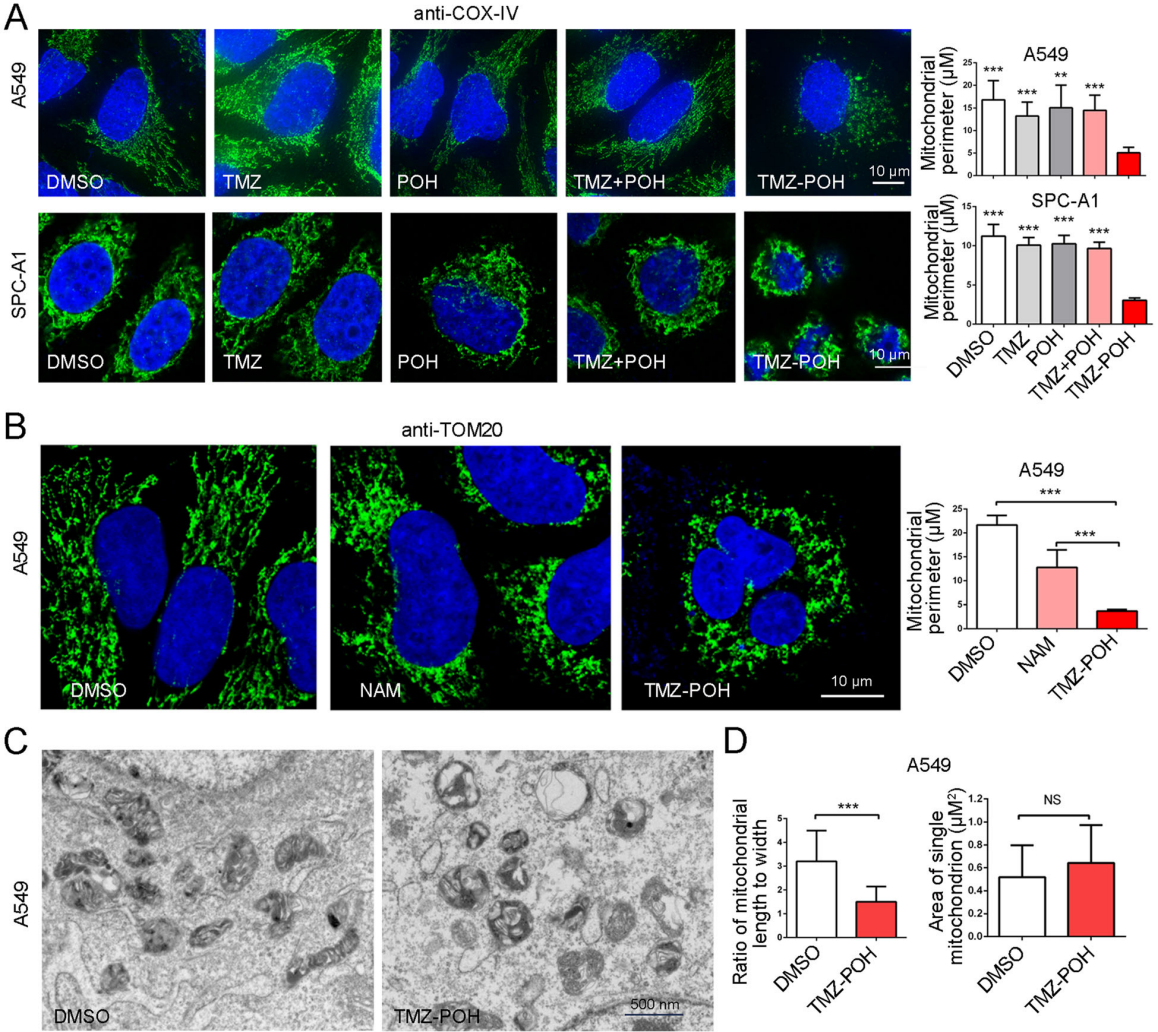
TMZ-POH leads to mitochondria fission. **a** A549, SPC-A1 cells treated with TMZ, POH, TMZ + POH, TMZ-POH respectively were fixed and stained with anti-COX-IV antibody. Mitochondrial perimeter per cell was measured using the Fiji Image J program. **b** A549 cells treated with NAM, TMZ-POH respectively were fixed and stained with anti-TOM20 antibody. Mitochondrial perimeter per cell was measured using the Fiji Image J program. **c** Mitochondria structure in A549 cells treated with TMZ-POH or DMSO was observed by TEM. **d** The shape and area of mitochondria in above drug-treated cells were measured using Fiji Image J program

Next, we checked the effect of TMZ-POH on mitochondrial fusion and fission. Immunostaining for COX-IV, a protein localized on the inner mitochondrial membrane was applied followed by treatment with TMZ-POH and its individual constituents. As shown in Fig. 2a, TMZ-POH induced accumulation of fragmented mitochondria with shorter lengths and fewer numbers of branches due to a lack of mitochondrial fusion whereas other drugs induced that of tubular mitochondria in A549 and SPC-A1 cells. Consistently, immunostaining used to elucidate the mitochondrial outer membranes (anti-TOM20) also demonstrated fragmented mitochondria accumulation in TMZ-POH treated group; Nevertheless, nicotinamide (NAM), an amide form of vitamin B3 can induce autophagy for clearing damaged mitochondria [23], induced tubular mitochondria accumulation (Fig. 2b), implying TMZ-POH unlike NAM might play an alternative role in mitochondrial clearance.

In addition, TEM observation showed that the morphology of mitochondria became more round and shorter, and the overall shapes of the mitochondria were grossly distorted, and the inner mitochondrial matrices were either severely damaged or almost absent in TMZ-POH group (Fig. 2c, and d, left), although their sizes seemed unchanged compared to the control group (Fig. 2d, right).

### TMZ-POH induces mitophagosome accumulation

**Fig. 3:**
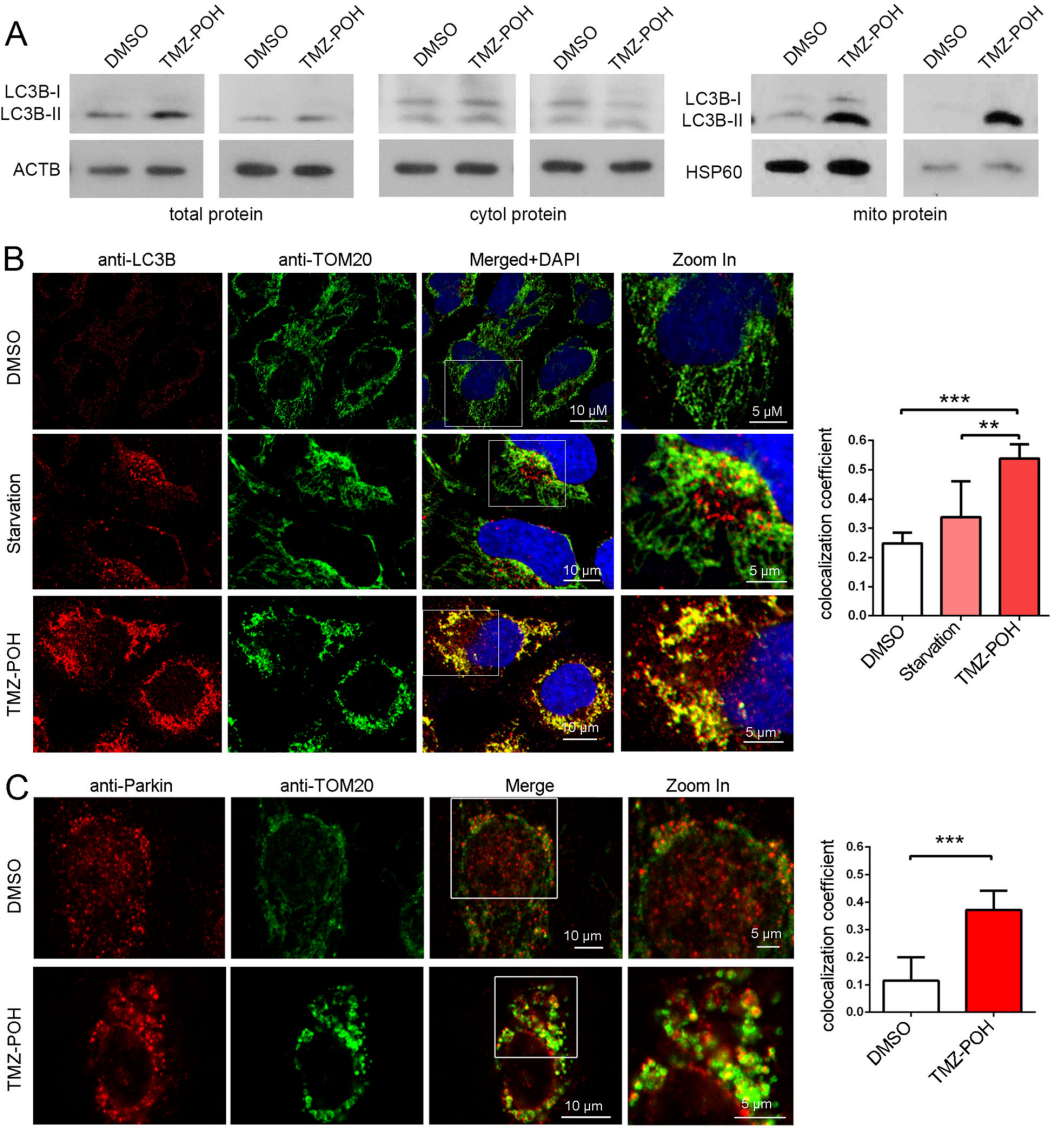
TMZ-POH induces mitophagosome accumulation. **a** Total protein, mitochondrial protein and cytoplasmic protein extracted from A549 and SPC-A1 cells were subjected to western blot analysis for demonstrating LC3B expression. ACTB acts as control for total and cytoplasmic protein, HSP60 acts as control for mitochondrial protein; (**b**) A549 cells were either starved or treated with TMZ-POH, and stained with anti-LC3B and anti-TOM20 antibodies and imaged by confocal microscopy; Statistical analysis of the colocalization coefficient of LC3B and TOM20 using Fiji Image J program; (**c**) A549 cells were treated with TMZ-POH or not, and stained with anti-Parkin and anti-TOM20 antibodies and imaged by confocal microscopy; Statistical analysis of the colocalization coefficient of Parkin and TOM20 using Fiji Image J program; The results shown are means ±SD, **p < 0.005, ***p < 0.001

It is well established that mitochondrial fission can promote mitophagy, a selective autophagy to remove damaged and dysfunctional mitochondria to protect the cells from excessive oxidative stress and cell death [24]. As shown in Fig. 3a and Additional file 2: Figure S2A, total protein, cytoplasmic protein and mitochondrial protein were collected and subjected to western blot analysis. TMZ-POH enhanced LC3B-II expression significantly in total and especially in mitochondrial protein and slightly in the cytoplasmic compared to control, indicating autophagosome formation induced by TMZ-POH almost occurred at mitochondria.

To further confirm this conclusion, colocalization of autophagosome (anti-LC3B) with mitochondria (anti-TOM20) was observed in A549 cells. Starvation as a non-selective autophagy inducer failed to improve the colocalization, while TMZ-POH significantly promoted this colocalization (Fig. 3b), which was also visualized and confirmed by TEM (Additional file 2: Figure S2B). Given that Parkin-ubiquitylated mitochondria is necessary for combination between mitochondrion and autophagosome [24], whether Parkin was localized at site of mitochondrion was detected. As shown in Fig. 3c, TMZ-POH promoted Parkin protein accumulated at mitochondria compared to control. Taken together, our data illuminate TMZ-POH induces mitophagosome accumulation.

### TMZ-POH blocks mitophagy flux

**Fig. 4:**
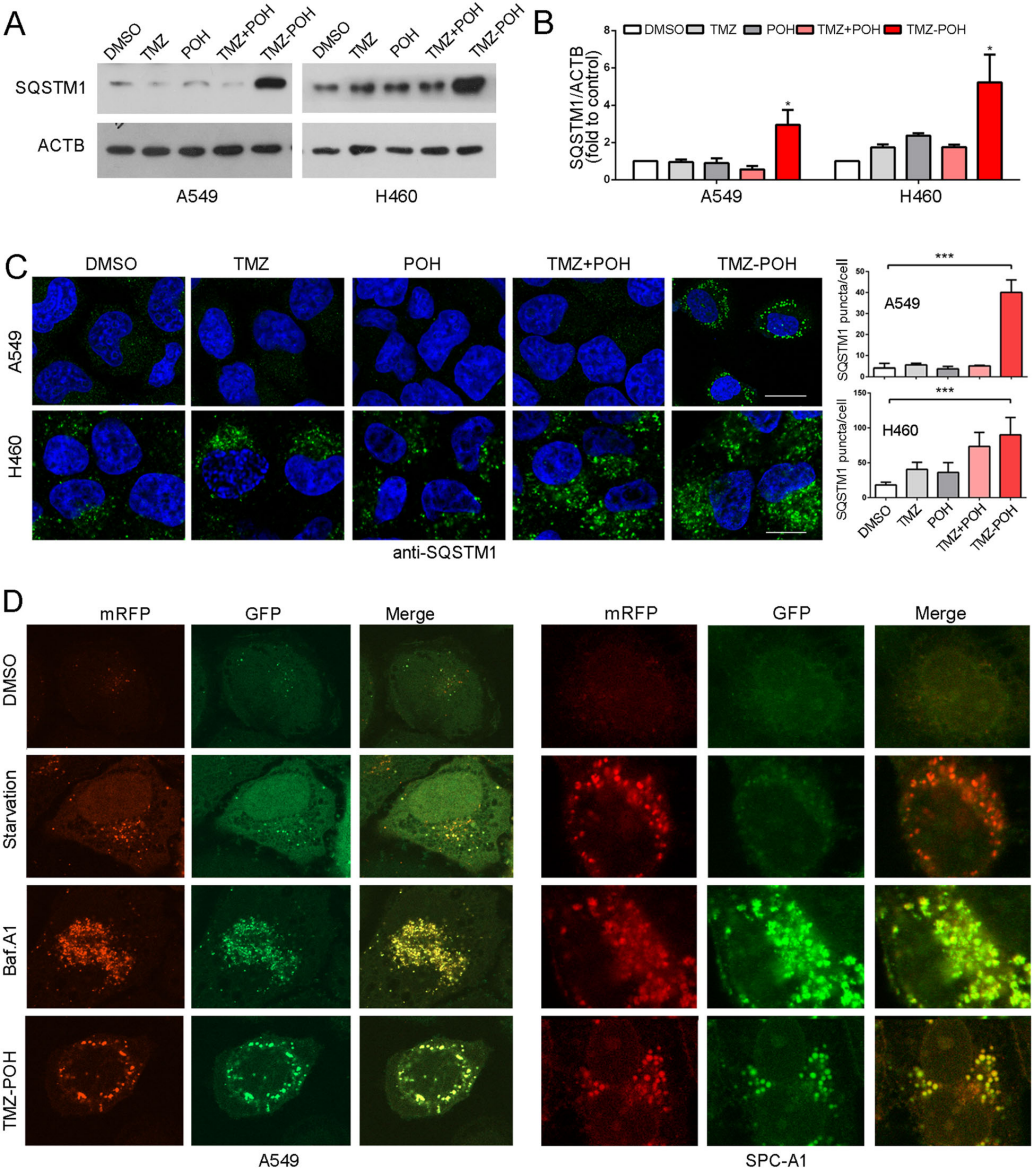
TMZ-POH blocks mitophagy flux. **a**-**c** Cells were treated with 100 μM TMZ, POH, TMZ + POH, TMZ-POH or DMSO respectively for 48 h in A549 and H460 cells. (A-B) Western blot analysis demonstrated SQSTM1 expression; (**c**) The above drug-treated cells were inspected under confocal laser microscopy to detect SQSTM1 puncta by immunofluorescence and SQSTM1 puncta number per cell was quantified using the Fiji Image J program. **d** A549 and SPC-A1 cells expressing mRFP-GFP-LC3 were starved or treated with Baf.A1 or TMZ-POH, and imaged by confocal microscopy. The results shown are means ±SD, *p < 0.05, **p < 0.005, ***p < 0.001

To validate the effect of TMZ-POH on mitophagy flux, the intracellular SQSTM1, a selective autophagic adaptor and incorporated with LC3B into autophagosomes and degraded by lysosomal hydrolyses [20], was firstly detected. We found TMZ-POH failed to degrade the SQSTM1 protein although it activated autophagosome formation significantly in A549 and H460 cells (Fig. 4a and b). Moreover, the immunostaining confirmed TMZ-POH upregulated SQSTM1 expression and promoted its assembling into aggregates (Fig. 4c).

**Fig. 5:**
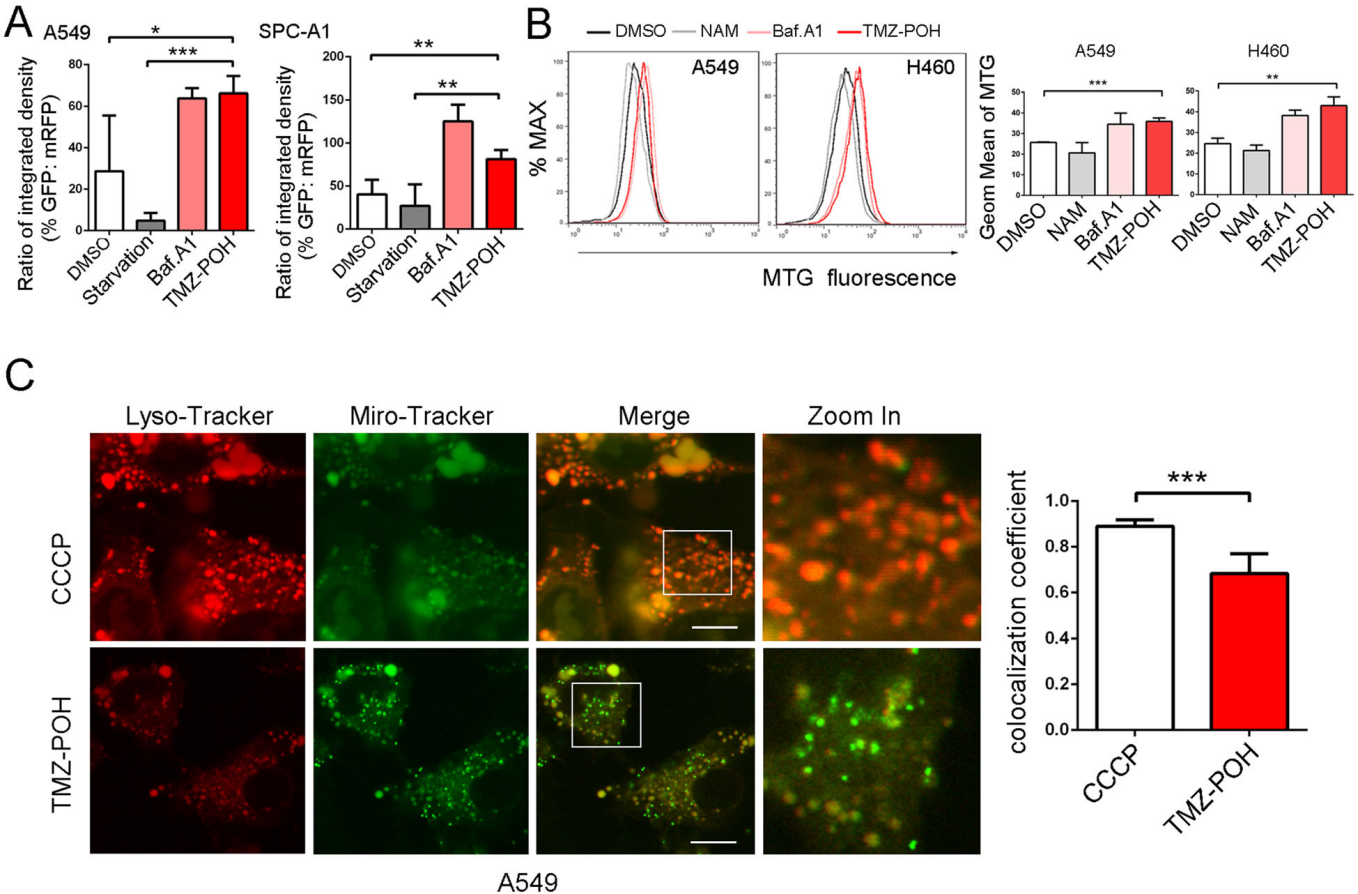
TMZ-POH impairs mitochondria elimination. **a** Statistical analysis of the percentage of punctate GFP signals that were positive for mRFP in A549 and SPC-A1 cells (**b**) A549 and H460 cells treated with NAM, Baf.A1 and TMZ-POH were loaded with MTG probe and subjected to flow cytometry. Mean fluorescence intensity (Geom Mean) of MTG was statistically analyzed. **c** A549 cells treated with CCCP or TMZ-POH were labelled with LTR and MTR and inspected under confocal laser microscopy. The colocalization coefficient of LTR and MTG was statistically analyzed. The results shown are means ±SD, *p < 0.05, **p < 0.005, ***p < 0.001

To explore the underlying mechanisms how TMZ-POH to block the mitophagy flux, we transfected A549 and SPC-A1 cells with the devised fusion protein mRFP-GFP-LC3 via adenovirus vector, which labels autophagosomes yellow because of superposition of GFP and mRFP signals, and autolysosomes red as the low lysosomal pH quenches the GFP signal [20]. As shown in Fig. 4d and Fig. 5a, most of the puncta lost the GFP signal and retained the mRFP signal in starved cells. However, in cells treated by TMZ-POH like Baf.A1 unlike those starved, quenching of the GFP was significantly diminished as indicated by the retention of both the mRFP and GFP signals.

In search of further evidence that TMZ-POH blocked mitophagy flux, mitochondrial population levels were determined by flow cytometry using Mito-Tracker Green (MTG) dye as described previously [25]. As shown in Fig. 5b, NAM induced a decrease of MTG fluorescence level, whereas TMZ-POH like Baf.A1 induced an increase of MTG fluorescence level relative to untreated cells. Besides, colocalization of lysosome (Lyso-Tracker Red, LTR) with mitochondria (MTG) was visualized in A549 cells. As shown in Fig. 5c, carbonyl cyanide m-chlorophenylhydrazone (CCCP), the most commonly used inducer of mitophagy [26], succeed to induce an overlap between MTG and LTR, whereas TMZ-POH failed, indicating mitophagy flux is blocked by TMZ-POH.

### TMZ-POH induces lysosomal dysfunction and decreases RAB7A expression

**Fig. 6:**
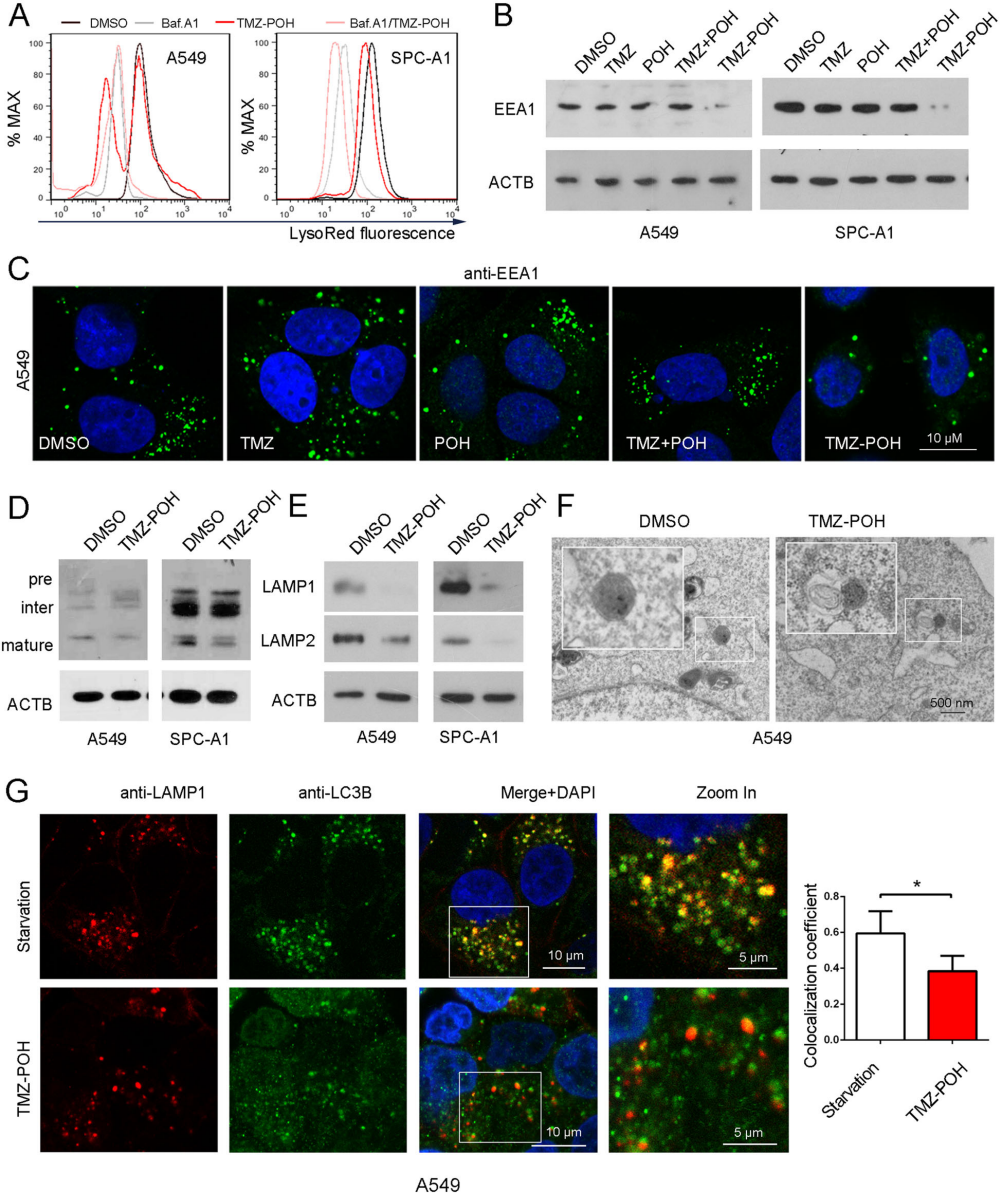
TMZ-POH impairs lysosome and its fusion with autophagosome (**a**) A549 and SPC-A1 cells were treated with TMZ-POH with or without presence of Baf.A1, and incubated with LTR and detected using flow cytometry; (**b**, **c**) A549 and SPC-A1 cells were treated with TMZ, POH, TMZ + POH, TMZ-POH or DMSO respectively, and were analyzed by either western blot (**b**) or immunostaining (**c**) using a specific EEA 1 antibody. **d** Western blot analysis of CTSD in A549 and SPC-A1 cells treated with TMZ-POH or not; (**e**) Western blot demonstrated LAMP1 and LAMP2 protein level in A549 and SPC-A1 cells treated with TMZ-POH or not; (**f**) Relative location of autophagosome and lysosome in A549 cells treated with TMZ-POH or DMSO was observed by TEM. **g** A549 cells were either starved or treated with TMZ-POH, and stained with anti-LAMP1 and anti-LC3B antibodies and imaged by confocal microscopy. Statistical analysis of the colocalization coefficient of LAMP1 and LC3B.The results shown are means ±SD, *p < 0.05

Since acidification is required for the maturation and activation of most lysosomal enzymes, the maintenance of acidity is a hallmark of functionally mature lysosomes [27]. We first stained A549 and SPC-A1 cells with LTR dye, which accumulates in acidic cell compartments, and acts as a lysosomal acidic indicator. This displayed a marked decrease in the LTR signal in response to TMZ-POH treatment, which was also observed in cells treated by Baf.A1 (Fig. 6a). As the lysosomal acidification depends on the process of the endocytic pathway [28], early endosome antigen 1 (EEA1) expression, a marker for early endosomes [29] was analyzed. Western blots and immunostaining showed TMZ-POH decreased down-regulated EEA1 expression as well as the early endosome formation significantly compared to other drugs (Fig. 6b, c, Additional file 3: Figure S3A, B). We further investigated the maturation of the lysosomal hydrolase cathepsin D (CTSD), which is synthesized in the endoplasmic reticulum, and finally matures in the lysosome to form 31-kDa and 14-kDa polypeptide [30]. We found that TMZ-POH clearly reduced the 31-kDa mature CTSD (Fig. 6d and Additional file 3: Figure S3C). In addition, LAMP 1 and 2, the important markers for mature lysosome [9], were also significantly downregulated by TMZ-POH, indicating altered endocytic trafficking or perturbed lysosomal maturation (Fig. 6e and Additional file 3: Figure. S3D).

We further investigated autophagosome-lysosome fusion via observing the colocalization of LC3B with LAMP1. As shown in Fig. 6g, starvation led to an overlap of LC3B with LAMP1, whereas TMZ-POH caused a significant decrease in the coefficient of LC3B-LAMP1 colocalization in A549. The impaired autophagosome-lysosome fusion was observed in TMZ-POH treated group but not in the untreated under TEM (Fig. 6f). Taken together, TMZ-POH impairs lysosome function and its fusion with autophagosome.

**Fig. 7:**
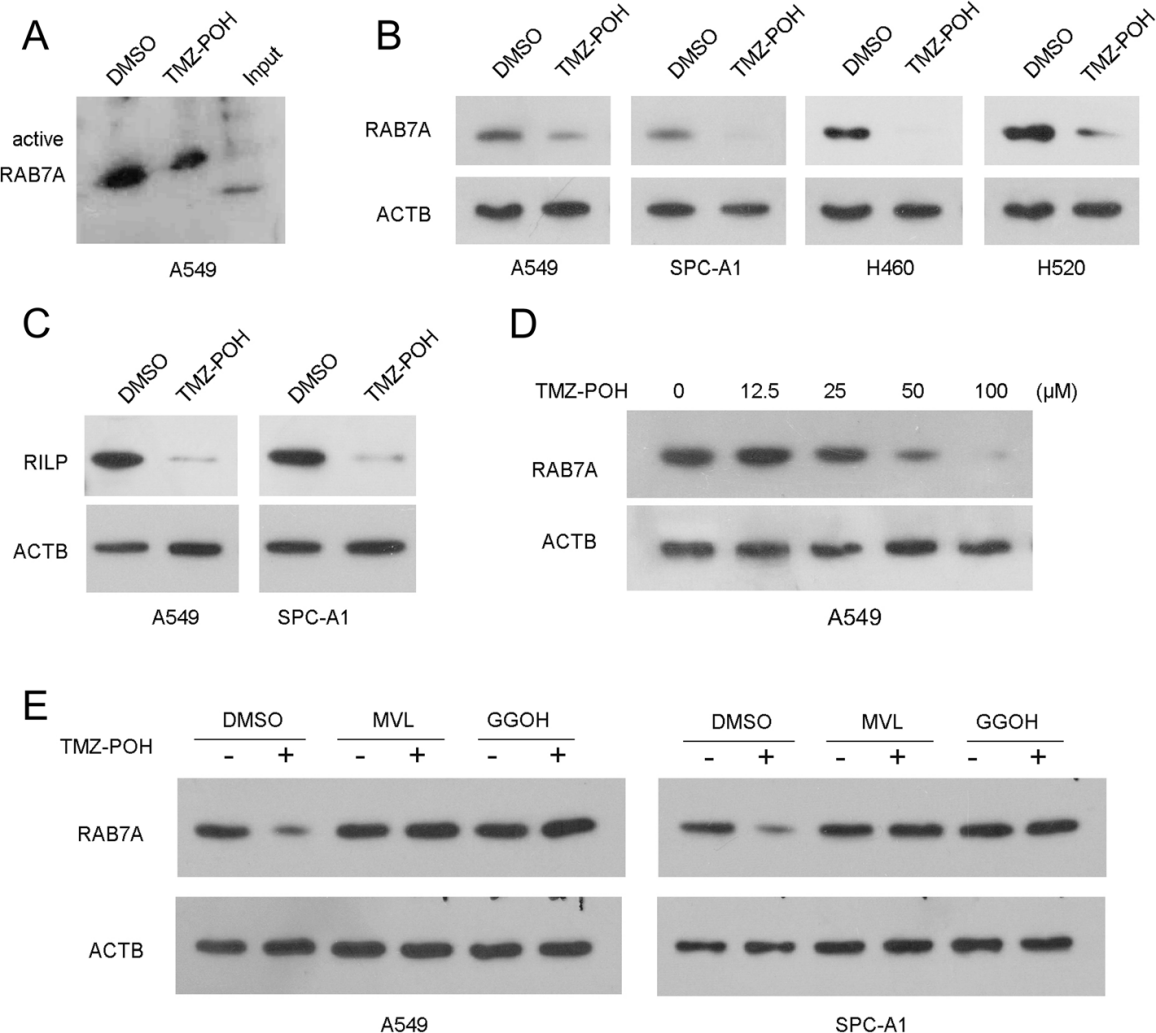
TMZ-POH down-regulates RAB7A via mevalonate pathway. **a** RAB7A activity was analyzed in lysates containing equal amounts of total proteins from A549 cells treated with TMZ-POH or not; (**b**, **c**) RAB7A expression in A549, SPC-A1, H460 and H520 cells (**b**); RILP expression in A549 and SPC-A1 cells (**c**) was detected by western blot when treated with TMZ-POH or not; (**d**) RAB7A in A549 treated with TMZ-POH for indicated concentration was analyzed by western blot; (**e**) A549 and SPC-A1 cells were treated with TMZ-POH with or without presence of MVL or GGOH, western blot analysis demonstrated RAB7A expression, ACTB as control

Given that RAB7A plays crucial roles in both endo-lysosomal maturation and autophagosome-lysosome fusion as described above, RAB7A activity was analyzed. TMZ-POH inhibited RAB7A activity significantly compared to control (Fig. 7a and Additional file 4: Figure S4A), and this inhibition largely resulted from the downregulation of TMZ-POH on RAB7A protein and its effector RILP expression significantly in all detected cells (Fig. 7b-c and Additional file 4: Figure S4B-C). Moreover, RAB7A protein was not affected at the low concentration (12.5 μM) of TMZ-POH, but suppressed significantly by high concentration (25, 50 and 100 μM) TMZ-POH in A549 cells, indicating TMZ-POH downregulated RAB7A dependent on concentration (Fig. 7d and Additional file 4: Figure S4D). RAB7A prenylation by geranylgeranyl transferase I and II (GGTT I/II) depended on mevalonate pathway, which contributes to its initial targeting to membranes for functioning [15]. Hence, we employed mevalonolactone (MVL) which was capable to hydrolyze to mevalonate [31] and geranylgeraniol (GGOH) [32], an intermediate of the mevalonate pathway upstream of RAB GGTase to co-incubate cells along with TMZ-POH. As shown in Fig. 7e and Additional file 4: Figure S4E, both MVL and GGOH were capable to restore TMZ-POH inhibited RAB7A protein. Taken together, our results indicate the downregulation of TMZ-POH on RAB7A might depend on mevalonate pathway.

### TMZ-POH induces apoptosis enhanced by 3-MA, and increases irradiation-induced apoptosis

**Fig. 8:**
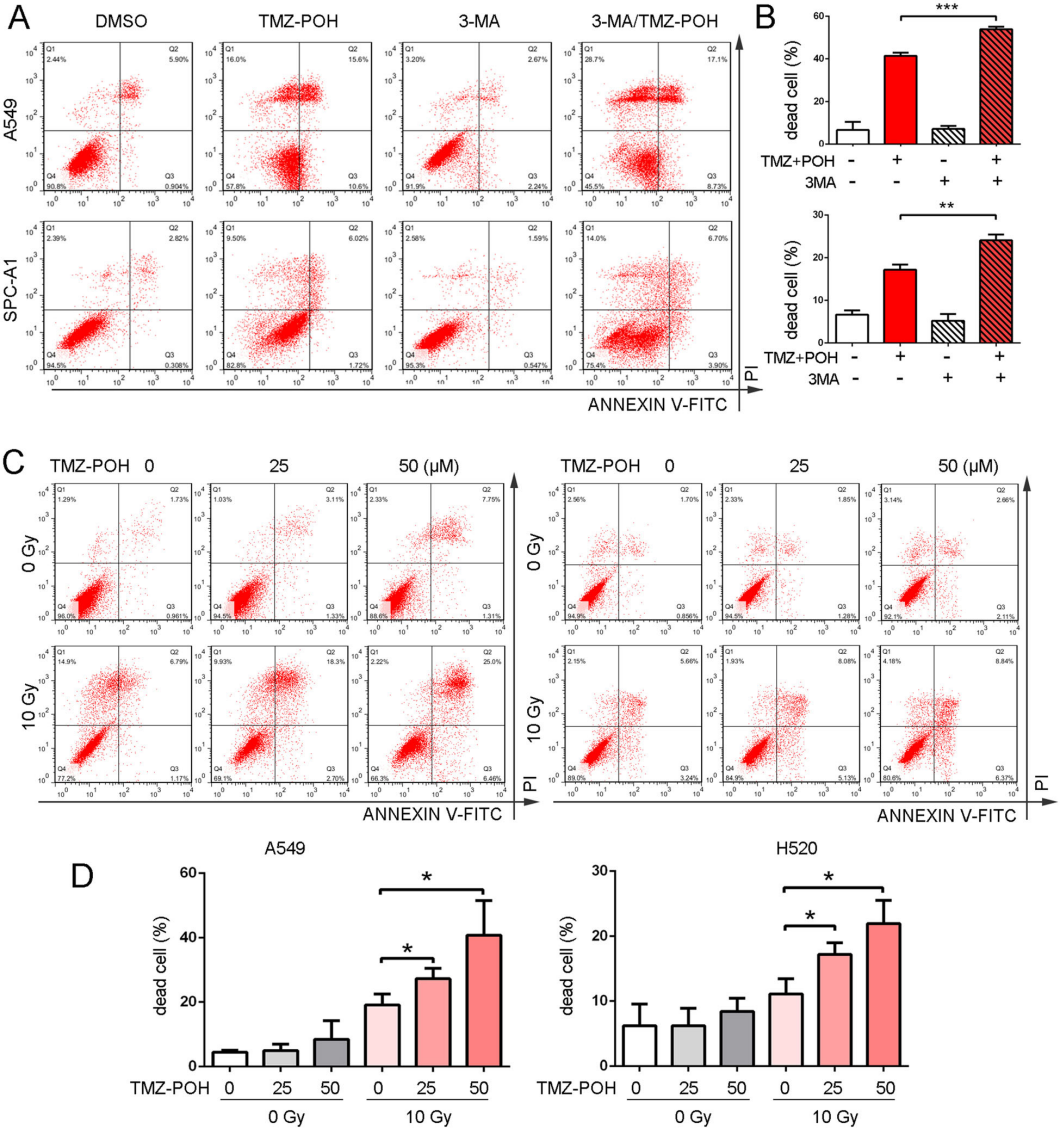
TMZ-POH induces apoptosis enhanced by 3-MA, and increases irradiation-induced apoptosis. **a** A549 and SPC-A1 cells were incubated with 3-MA (1 mM) combined with TMZ-POH (100 μM) for 48 h, and finally subjected to apoptosis analysis; **b** The percent of the dead cells was statistically analyzed. **c** A549 and H520 cells were subjected to 10 Gy irradiation for 72 h, and were treated with 25 or 50 μM TMZ-POH during the last 48 h, an apoptosis analysis was applied; **d** The percent of the dead cells was statistically analyzed. The results shown are means ±SD, *p < 0.05, **p < 0.005, ***p < 0.001

Previous study has reported the antitumor effect of TMZ was suppressed when autophagy was prevented by VPS34 inhibitor 3-methyladenine (3-MA) to reduce the formation of autophagosomes [33]. Our results showed that TMZ-POH induced obvious apoptosis both in A549 and SPC-A1 cells coincident with our previous studies [4, 5], which was significantly enhanced by 3-MA mediated autophagy inhibition (Fig. 8a-b), indicating inhibition of autophagosome formation failed to protect cells against TMZ-POH-induced cell death, which was not an “autophagic cell death”.

It has been well established autophagy acts as recycling and defensive mechanisms to supply cells and protect them from cell death. Blockage of autophagic flux surely lead to an insufficient energy supply and sensitizing cells to external stimuli. Whether autophagic flux blockage induced by TMZ-POH sensitized cancer cells to death was also verified. A549 and H520 cells were subjected to irradiation first to stimulate cell death, which also was able to induce autophagy and be sensitized when autophagy inhibited [34]. As shown in Fig. 8c-d, an apoptosis assay revealed that 10 Gy irradiation increased cell apoptosis after 72 h of treatment, and treatment of cells with 25 or 50 μM TMZ-POH during the last 48 h further increased cell apoptosis. These results suggest that treatment of cancer cells with TMZ-POH blocks the activation of autophagic flux by irradiation and increases the sensitivity to the cytotoxic action of irradiation, thus proposing TMZ-POH as a potential radiosensitizer.

## Discussion

In the present study, we demonstrated the relationship between TMZ-POH and autophagy/mitophagy. Our results showed that TMZ-POH suppressed rather than stimulated mitophagy. TMZ-POH impaired lysosomal acidification and maturation, and hampered autophagosome-lysosome fusion, resulting in mitophagosomes accumulation. Furthermore, our data suggested TMZ-POH downregulated small GTPase RAB7A expression via mevalonate pathway, which was involved in the process TMZ-POH blocked mitophagy flux.

Nowadays, the association between TMZ and autophagy has been clarified [16, 17], TMZ induces the sustained inhibition of AKT-mTOR, and in turn produces an induction of autophagy [35] in glioma, indicating TMZ-induced autophagy depends on mTOR signaling. However, TMZ failed to affect the mTOR signaling and to induce autophagy in NSCLC cells, this is probably caused by tissue specificity or different drug treated time and concentration. Besides, we didn’t rule out the role of TMZ-POH in the autophagy induction although it didn’t affect mTOR signaling either. In our previous study, TMZ-POH induced ROS accumulation in NSCLC cells [4], and we believed that it induced ROS accumulation also contributed to mitochondria fission and autophagy. In this study, TMZ-POH induced autophagosome accumulation might attribute to its blockage of autophagic degradation, the late stage of autophagy flux.

Our data suggest at least an involvement of an inhibitory effect of TMZ-POH at the late stage of autophagy. Several lines of evidence validate this conclusion. First, TMZ-POH arrested the degradation of LC3B and SQSTM1, the selective autophagy substrates, and led to increased accumulation of Mito-Tracker signaling, mimicking the action of Baf. A1; Second, TMZ-POH impaired lysosomal acidification, as well as hampered lysosomal maturation; Next, TMZ-POH blocked the fusion of lysosomes with autophagosomes; Finally, TMZ-POH down-regulated RAB7A expression, which plays crucial roles in both endo-lysosomal maturation and autophagosome-lysosome fusion. Notably, the regulation of TMZ-POH on RAB7A quite differed from its POH component. POH, an inhibitor of prenyltransferases, is believed to inactivate RAB GTPases protein by impairing its prenylation, while POH did not change their protein levels [36], indicating POH suppressed Rab GTPases activity independent on down-regulating these protein. In contrast, TMZ-POH downregulated RAB7A significantly at the protein, as well as it also led to an obvious decrease in RAB7A activity.

Until recently, the detailed relationship between autophagy and apoptosis remains unclear. In our study, 3-MA failed to exert its protective effect on TMZ-POH induced apoptosis but facilitate it, indicating TMZ-POH induced apoptosis was independent of autophagosome formation. Treatment with combination of TMZ-POH and 3-MA led to a more complete dual inhibition at both the early by 3-MA and late stage by TMZ-POH in autophagy. More importantly, our data demonstrated TMZ-POH was capable to sensitize cancer cell to irradiation induced apoptosis, which was largely due to its impact on autophagic flux. As a cell death executor, mitochondria are now recognized to play an important role in radiation induced cellular responses [37], radiation alters mitochondrial functions, increases mitochondrial oxidative stress, induces apoptosis and causes mitochondrial DNA (mtDNA) damage [38]. Mitochondrial damage could induce mitophagy to control mitochondrial number and quality, which is capable to segregate and degrade dysfunctional mitochondria that might otherwise release ROS, pro-apoptotic proteins, and other toxic mediators, plays a protective role. In current study, we demonstrated TMZ-POH impaired mitophagic flux, hindered the degradation and elimination recycling of toxic products of irradiation, and facilitated irradiation induced apoptosis, thereby proposing TMZ-POH as a potential radiosensitizer.

## Conclusion

**Fig. 9:**
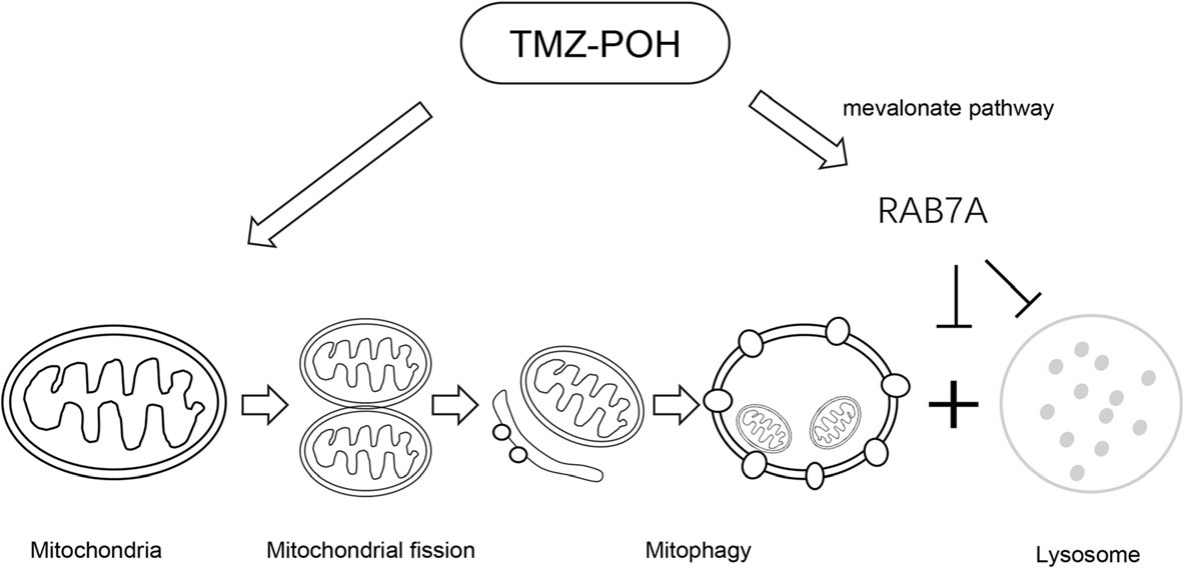
Overview of pathways for TMZ-POH impaired mitophagic flux. Although TMZ-POH induced mitochondrial fission and autophagosome accumulation which colocalized with mitochondria in the cells significantly, it inclined to block mitophagy flux via inducing lysosomal dysfunction and hampered autophagosome-lysosome fusion due to its downregulation on the small GTPase RAB7A via mevalonate pathway

In summary, as shown in Fig. 9, although TMZ-POH induced mitochondrial fission and autophagosome accumulation which colocalized with mitochondria in the cells significantly, it inclined to block mitophagy flux via inducing lysosomal dysfunction and hampered autophagosome-lysosome fusion due to its downregulation on the small GTPase RAB7A via mevalonate pathway. More importantly, TMZ-POH was capable to sensitize cancer cell to irradiation induced apoptosis, thereby proposing TMZ-POH as a potential radiosensitizer.

## Additional files

Additional file 1: **Figure S1.** (A) Cells were treated with 100 μM TMZ, POH, TMZ + POH, TMZ-POH or DMSO respectively for 48 h. The LC3B-II expression in above drug-treated A549, SPC-A1, H460 and H520 cells were statistically analyzed. (B) SPC-A1 cells were treated with indicated concentration of TMZ-POH, and were inspected under confocal laser microscopy to detect LC3B puncta by immunofluorescence. LC3B puncta number per cell was quantified using the Fiji Image J program. (C) SPC-A1 cells treated with 100 μM TMZ-POH or DMSO were using western blot to detect LC3B and ACTB expression in the presence or absence of Baf.A1. The LC3B-II expression was statistically analyzed. (D-E) SPC-A1 and H460 cells were treated with 100 μM TMZ, POH, TMZ + POH, TMZ-POH or DMSO respectively for 48 h, western blot demonstrated BECN1, pho-mTOR, mTOR and pho-P70S6K expression. The results shown are means ±SD, *p < 0.05, **p < 0.005, NS = no significance. (JPG 1143 kb)

Additional file 2: **Figure S2.** (A) The LC3B-II expression of total protein, mitochondrial protein and cytoplasmic protein extracted from A549 and SPC-A1 cells when treated with TMZ-POH or not were statistically analyzed. (B) Mitophagosomes were observed by TEM in A549 treated with TMZ-POH. The results shown are means ±SD, **p < 0.005, ***p < 0.001, NS = no significance. (JPG 567 kb)

Additional file 3: **Figure S3.** (A) The EEA1 expression in A549 and SPC-A1 cells when treated with TMZ-POH or not was statistically analyzed. (B) The EEA1 puncta number per cell was quantified and statistically analyzed. (C) The mature CTSD expression in A549 and SPC-A1 cells when treated with TMZ-POH or not was statistically analyzed. (D) The LAMP1 and LAMP2 expression in A549 and SPC-A1 cells when treated with TMZ-POH or not were statistically analyzed. The results shown are means ±SD, **p < 0.005, ***p < 0.001. (JPG 457 kb)

Additional file 4: **Figure S4.** (A) RAB7A activity of A549 cells treated with TMZ-POH or not was statistically analyzed. (B) The RAB7A expression in A549, SPC-A1, H460 and H520 cells when treated with TMZ-POH or not was statistically analyzed. (C) The RILP expression in A549 and SPC-A1 cells when treated with TMZ-POH or not was statistically analyzed. (D) The RAB7A expression in A549 cells treated with TMZ-POH for indicated concentration was statistically analyzed. (E) The RAB7A expression in A549 and SPC-A1 cells when treated with TMZ-POH with or without presence of MVL or GGOH was statistically analyzed. The results shown are means ±SD, *p < 0.05, **p < 0.005, ***p < 0.001, NS = no significance. (JPG 755 kb)

## Abbreviations

3-MA: 3-methyladenine
Baf.A1: Bafilomycin A1
BECN1: Beclin 1
CCCP: Carbonyl cyanide m-chlorophenylhydrazone
CTSD: Cathepsin D
EEA1: Early endosome antigen 1
GGOH: Geranylgeraniol
GGTT I/II: Geranylgeranyl transferase I and II
LAMP: Lysosome-associated membrane protein
LTR: Lyso-Tracker Red
MTG: Mito-Tracker Green
mTOR: Mammalian target of rapamycin
MVL: Mevalonolactone
NAM: Nicotinamide
NPC: Nasopharyngeal carcinoma
NSCLC: Non-small cell lung cancer
P70S6K: p70 S6 kinase
POH: Perillyl alcohol
RAB7A: Ras-associated binding protein 7A
ROS: Reactive oxygen species
TEM: Transmission electron microscope
TMZ: Temozolomide
TMZ-POH: Temozolomide-perillyl alcohol conjugate;
TNBC: Triple-negative breast cancer

## References

[1] Cho HY, Wang W, Jhaveri N, Torres S, Tseng J, Leong MN, Lee DJ, Goldkorn A, Xu T, Petasis NA (2012). Perillyl alcohol for the treatment of temozolomide-resistant gliomas. Mol Cancer Ther.

[2] Cho HY, Wang W, Jhaveri N, Lee DJ, Sharma N, Dubeau L, Schonthal AH, Hofman FM, Chen TC (2014). NEO212, temozolomide conjugated to perillyl alcohol, is a novel drug for effective treatment of a broad range of temozolomide-resistant gliomas. Mol Cancer Ther.

[3] Chen TC, Cho HY, Wang W, Barath M, Sharma N, Hofman FM, Schonthal AH (2014). A novel temozolomide-perillyl alcohol conjugate exhibits superior activity against breast cancer cells in vitro and intracranial triple-negative tumor growth in vivo. Mol Cancer Ther.

[4] Song X, Xie L, Wang X, Zeng Q, Chen TC, Wang W, Song X (2016). Temozolomide-perillyl alcohol conjugate induced reactive oxygen species accumulation contributes to its cytotoxicity against non-small cell lung cancer. Sci Rep.

[5] Xie L, Song X, Guo W, Wang X, Wei L, Li Y, Lv L, Wang W, Chen TC, Song X (2016). Therapeutic effect of TMZ-POH on human nasopharyngeal carcinoma depends on reactive oxygen species accumulation. Oncotarget.

[6] Kim B, Song YS (2016). Mitochondrial dynamics altered by oxidative stress in cancer. Free Radic Res.

[7] Sena LA, Chandel NS (2012). Physiological roles of mitochondrial reactive oxygen species. Mol Cell.

[8] Shen HM, Mizushima N (2014). At the end of the autophagic road: an emerging understanding of lysosomal functions in autophagy. Trends Biochem Sci.

[9] Huynh KK, Eskelinen EL, Scott CC, Malevanets A, Saftig P, Grinstein S (2007). LAMP proteins are required for fusion of lysosomes with phagosomes. EMBO J.

[10] Wang T, Ming Z, Xiaochun W, Hong W (2011). Rab7: role of its protein interaction cascades in endo-lysosomal traffic. Cell Signal.

[11] Poteryaev D, Datta S, Ackema K, Zerial M, Spang A (2010). Identification of the switch in early-to-late endosome transition. Cell.

[12] Jordens I, Fernandez-Borja M, Marsman M, Dusseljee S, Janssen L, Calafat J, Janssen H, Wubbolts R, Neefjes J (2001). The Rab7 effector protein RILP controls lysosomal transport by inducing the recruitment of dynein-dynactin motors. Curr Biol.

[13] Zhang M, Chen L, Wang S, Wang T (2009). Rab7: roles in membrane trafficking and disease. Biosci Rep.

[14] Schafer WR, Rine J (1992). Protein prenylation: genes, enzymes, targets, and functions. Annu Rev Genet.

[15] Shinde SR, Maddika S. Post translational modifications of Rab GTPases. Small GTPases. 2017:1–8. 10.1080/21541248.2017.1299270 PMC5902199 28426288

[16] Kanzawa T, Germano IM, Komata T, Ito H, Kondo Y, Kondo S (2004). Role of autophagy in temozolomide-induced cytotoxicity for malignant glioma cells. Cell Death Differ.

[17] Natsumeda M, Aoki H, Miyahara H, Yajima N, Uzuka T, Toyoshima Y, Kakita A, Takahashi H, Fujii Y (2011). Induction of autophagy in temozolomide treated malignant gliomas. Neuropathology.

[18] Shacka JJ, Klocke BJ, Roth KA (2006). Autophagy, bafilomycin and cell death: the "a-B-cs" of plecomacrolide-induced neuroprotection. Autophagy.

[19] Schindelin J, Arganda-Carreras I, Frise E, Kaynig V, Longair M, Pietzsch T, Preibisch S, Rueden C, Saalfeld S, Schmid B (2012). Fiji: an open-source platform for biological-image analysis. Nat Methods.

[20] Klionsky DJ, Abdelmohsen K, Abe A, Abedin MJ, Abeliovich H, Acevedo Arozena A, Adachi H, Adams CM, Adams PD, Adeli K (2016). Guidelines for the use and interpretation of assays for monitoring autophagy (3rd edition). Autophagy.

[21] Laplante M, Sabatini DM (2012). mTOR signaling in growth control and disease. Cell.

[22] Puente C, Hendrickson RC, Jiang X (2016). Nutrient-regulated phosphorylation of ATG13 inhibits starvation-induced autophagy. J Biol Chem.

[23] Jang SY, Kang HT, Hwang ES (2012). Nicotinamide-induced mitophagy: event mediated by high NAD+/NADH ratio and SIRT1 protein activation. J Biol Chem.

[24] Ashrafi G, Schwarz TL (2013). The pathways of mitophagy for quality control and clearance of mitochondria. Cell Death Differ.

[25] Xiao B, Deng X, Zhou W, Tan EK (2016). Flow cytometry-based assessment of Mitophagy using MitoTracker. Front Cell Neurosci.

[26] Narendra D, Tanaka A, Suen DF, Youle RJ (2008). Parkin is recruited selectively to impaired mitochondria and promotes their autophagy. J Cell Biol.

[27] Mindell JA (2012). Lysosomal acidification mechanisms. Annu Rev Physiol.

[28] Saftig P: Physiology of the lysosome. In Fabry Disease: Perspectives from 5 Years of FOS. Edited by Mehta A, Beck M, Sunder-Plassmann G. Oxford; 2006. 21290683

[29] Mu FT, Callaghan JM, Steele-Mortimer O, Stenmark H, Parton RG, Campbell PL, McCluskey J, Yeo JP, Tock EP, Toh BH (1995). EEA1, an early endosome-associated protein. EEA1 is a conserved alpha-helical peripheral membrane protein flanked by cysteine "fingers" and contains a calmodulin-binding IQ motif. J Biol Chem.

[30] Cai Q, Lu L, Tian JH, Zhu YB, Qiao H, Sheng ZH (2010). Snapin-regulated late endosomal transport is critical for efficient autophagy-lysosomal function in neurons. Neuron.

[31] Lacher SM, Bruttger J, Kalt B, Berthelet J, Rajalingam K, Wortge S, Waisman A (2017). HMG-CoA reductase promotes protein prenylation and therefore is indispensible for T-cell survival. Cell Death Dis.

[32] Schumacher MM, Elsabrouty R, Seemann J, Jo Y, DeBose-Boyd RA. The prenyltransferase UBIAD1 is the target of geranylgeraniol in degradation of HMG CoA reductase. Elife. 2015;4. 10.7554/eLife.05560 PMC4374513 25742604

[33] Wu YT, Tan HL, Shui G, Bauvy C, Huang Q, Wenk MR, Ong CN, Codogno P, Shen HM (2010). Dual role of 3-methyladenine in modulation of autophagy via different temporal patterns of inhibition on class I and III phosphoinositide 3-kinase. J Biol Chem.

[34] Ito H, Daido S, Kanzawa T, Kondo S, Kondo Y (2005). Radiation-induced autophagy is associated with LC3 and its inhibition sensitizes malignant glioma cells. Int J Oncol.

[35] Filippi-Chiela EC, Bueno e Silva MM, Thome MP, Lenz G (2015). Single-cell analysis challenges the connection between autophagy and senescence induced by DNA damage. Autophagy.

[36] Ren Z, Elson CE, Gould MN (1997). Inhibition of type I and type II geranylgeranyl-protein transferases by the monoterpene perillyl alcohol in NIH3T3 cells. Biochem Pharmacol.

[37] Zhang B, Davidson MM, Zhou H, Wang C, Walker WF, Hei TK (2013). Cytoplasmic irradiation results in mitochondrial dysfunction and DRP1-dependent mitochondrial fission. Cancer Res.

[38] Kam WW, Banati RB (2013). Effects of ionizing radiation on mitochondria. Free Radic Biol Med.

