# Supplementary figures and images for: Temozolomide-Perillyl alcohol conjugate impairs Mitophagy flux by inducing lysosomal dysfunction in non-small cell lung Cancer cells and sensitizes them to irradiation
Source: J Exp Clin Cancer Res. 2018 Oct 16;37:250. doi: 10.1186/s13046-018-0905-1 (PMC6191917; doi:10.1186/s13046-018-0905-1)

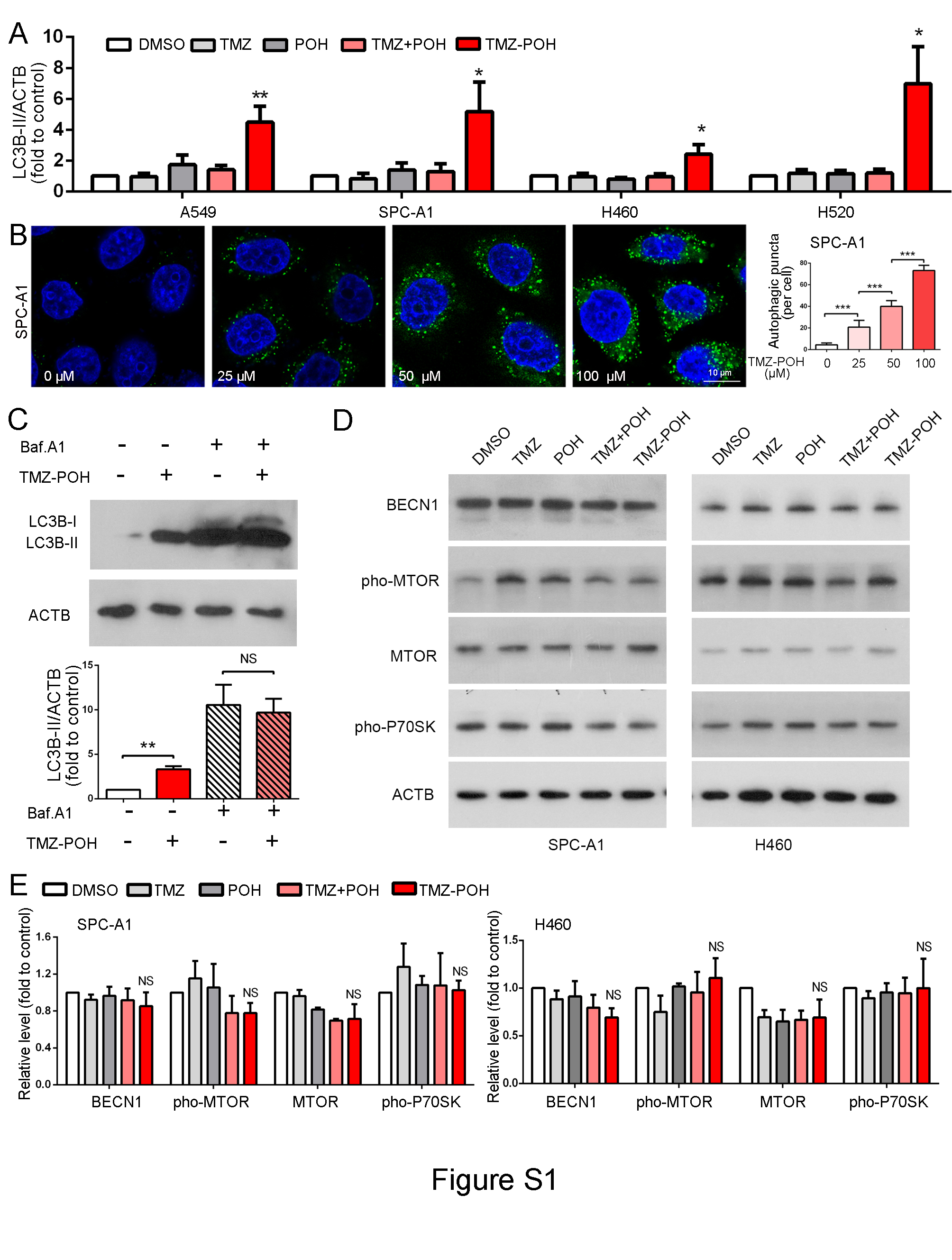

Supplement: Supplementary file 1 — Figure S1. (A) Cells were treated with 100 μM TMZ, POH, TMZ + POH, TMZ-POH or DMSO respectively for 48 h. The LC3B-II expression in above drug-treated A549, SPC-A1, H460 and H520 cells were statistically analyzed. (B) SPC-A1 cells were treated with indicated concentration of TMZ-POH, and were inspected under confocal laser microscopy to detect LC3B puncta by immunofluorescence. LC3B puncta number per cell was quantified using the Fiji Image J program. (C) SPC-A1 cells treated with 100 μM TMZ-POH or DMSO were using western blot to detect LC3B and ACTB expression in the presence or absence of Baf.A1. The LC3B-II expression was statistically analyzed. (D-E) SPC-A1 and H460 cells were treated with 100 μM TMZ, POH, TMZ + POH, TMZ-POH or DMSO respectively for 48 h, western blot demonstrated BECN1, pho-mTOR, mTOR and pho-P70S6K expression. The results shown are means ±SD, *p < 0.05, **p < 0.005, NS = no significance. (JPG 1143 kb) [file 13046_2018_905_MOESM1_ESM.jpg]

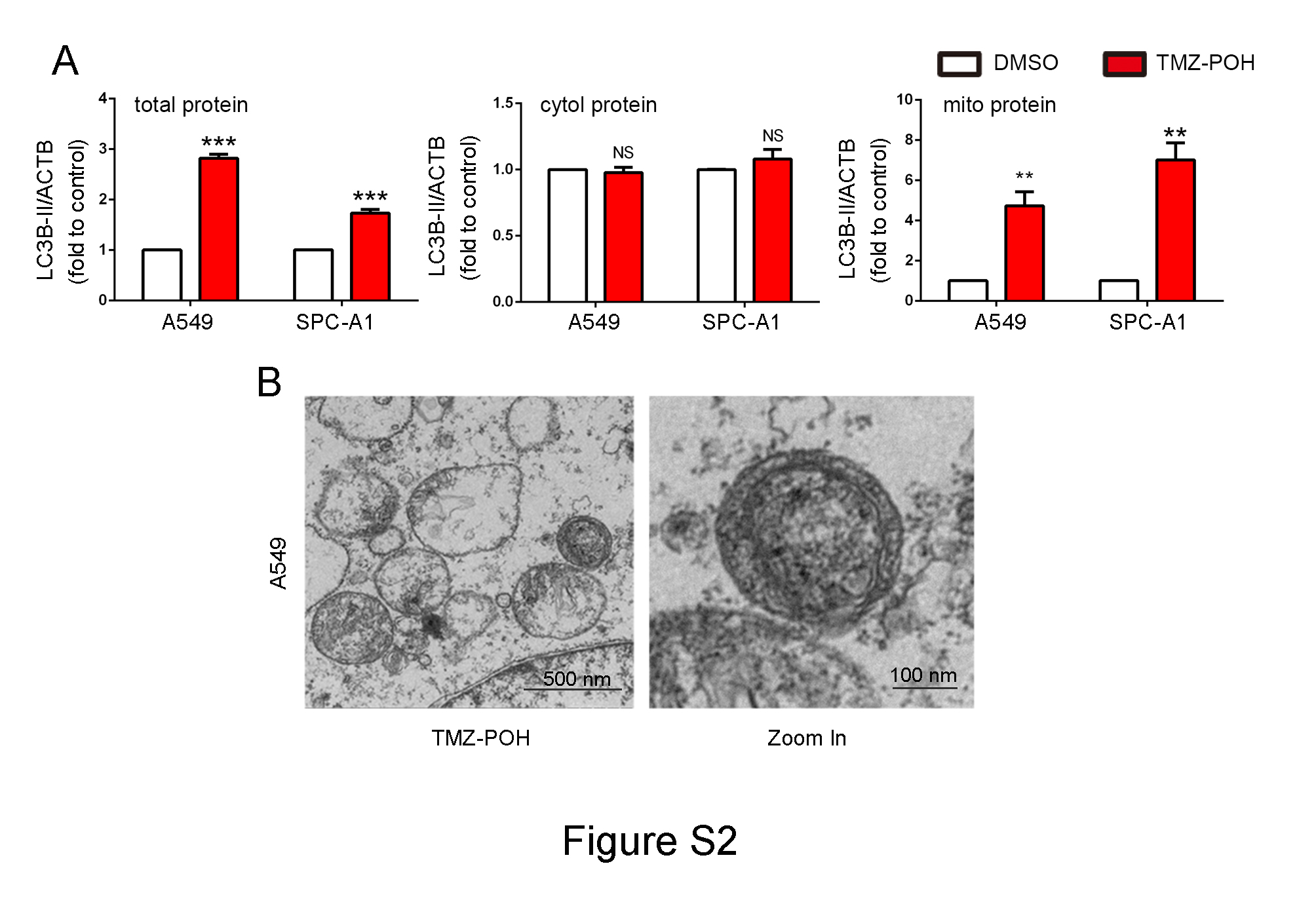

Supplement: Supplementary file 2 — Figure S2. (A) The LC3B-II expression of total protein, mitochondrial protein and cytoplasmic protein extracted from A549 and SPC-A1 cells when treated with TMZ-POH or not were statistically analyzed. (B) Mitophagosomes were observed by TEM in A549 treated with TMZ-POH. The results shown are means ±SD, **p < 0.005, ***p < 0.001, NS = no significance. (JPG 567 kb) [file 13046_2018_905_MOESM2_ESM.jpg]

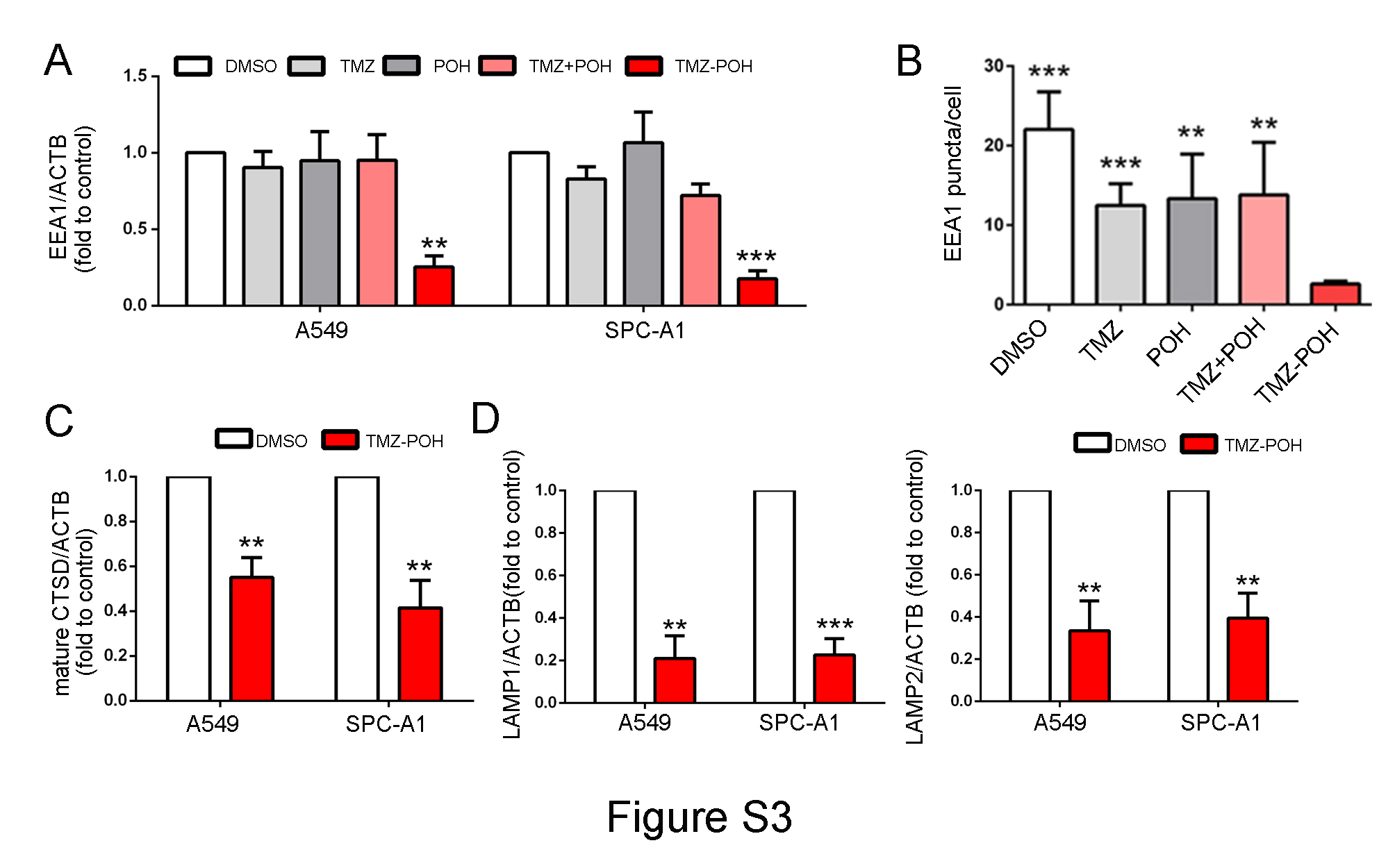

Supplement: Supplementary file 3 — Figure S3. (A) The EEA1 expression in A549 and SPC-A1 cells when treated with TMZ-POH or not was statistically analyzed. (B) The EEA1 puncta number per cell was quantified and statistically analyzed. (C) The mature CTSD expression in A549 and SPC-A1 cells when treated with TMZ-POH or not was statistically analyzed. (D) The LAMP1 and LAMP2 expression in A549 and SPC-A1 cells when treated with TMZ-POH or not were statistically analyzed. The results shown are means ±SD, **p < 0.005, ***p < 0.001. (JPG 457 kb) [file 13046_2018_905_MOESM3_ESM.jpg]

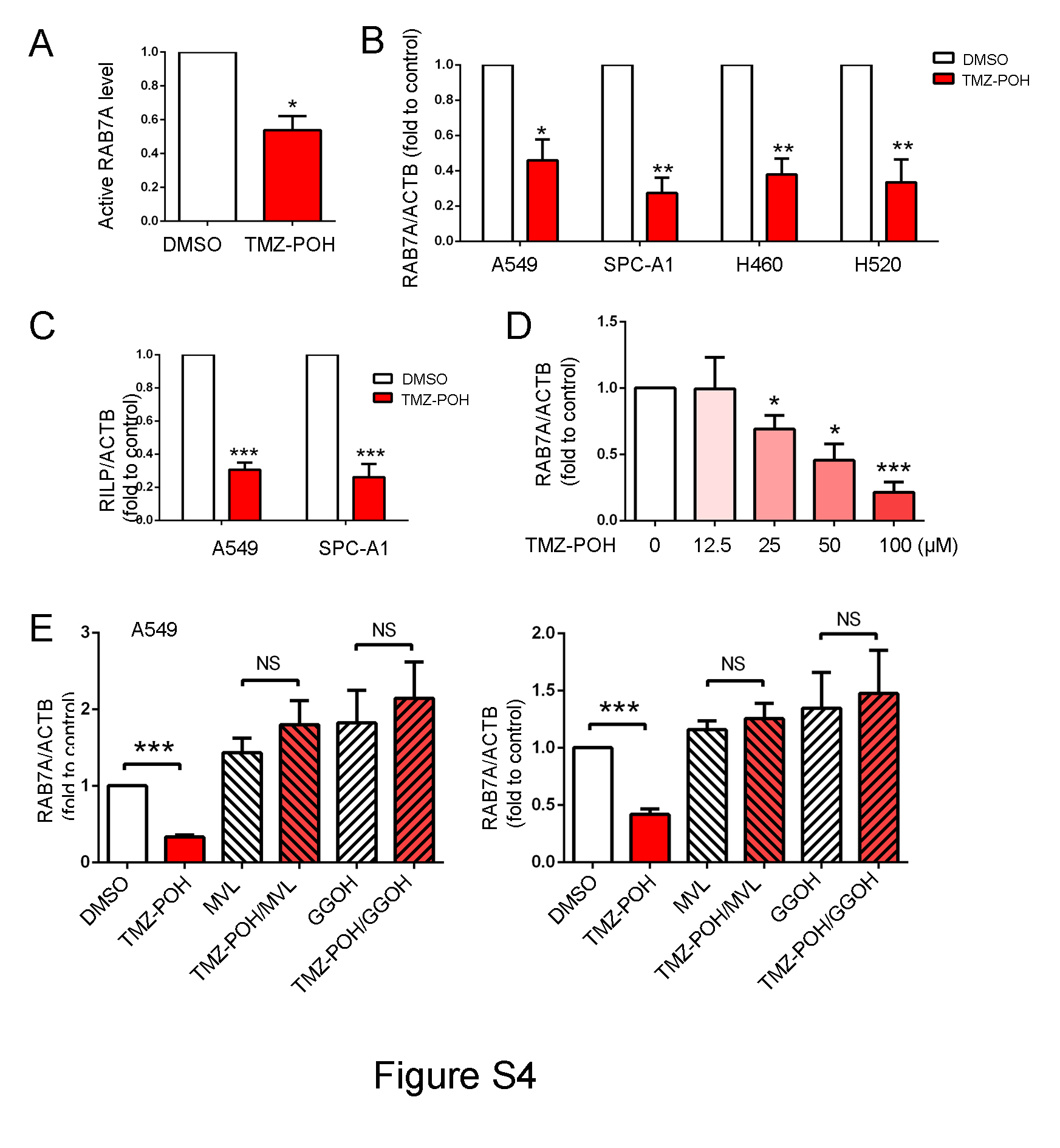

Supplement: Supplementary file 4 — Figure S4. (A) RAB7A activity of A549 cells treated with TMZ-POH or not was statistically analyzed. (B) The RAB7A expression in A549, SPC-A1, H460 and H520 cells when treated with TMZ-POH or not was statistically analyzed. (C) The RILP expression in A549 and SPC-A1 cells when treated with TMZ-POH or not was statistically analyzed. (D) The RAB7A expression in A549 cells treated with TMZ-POH for indicated concentration was statistically analyzed. (E) The RAB7A expression in A549 and SPC-A1 cells when treated with TMZ-POH with or without presence of MVL or GGOH was statistically analyzed. The results shown are means ±SD, *p < 0.05, **p < 0.005, ***p < 0.001, NS = no significance. (JPG 755 kb) [file 13046_2018_905_MOESM4_ESM.jpg]
